# A critical appraisal on AIT in childhood asthma

**DOI:** 10.1186/s12948-018-0085-8

**Published:** 2018-03-06

**Authors:** Matteo Ferrando, Francesca Racca, Lorena Nascimento Girardi Madeira, Enrico Heffler, Giovanni Passalacqua, Francesca Puggioni, Niccolò Stomeo, Giorgio Walter Canonica

**Affiliations:** 1 Allergy & Respiratory Diseases, DIMI Dept of Internal Medicine, University of Genoa, IRCCS AOU San Martino-IST, Genoa, Italy; 2 Personalized Medicine, Asthma and Allergy Clinic, Humanitas University and Research Hospital, Via Alessandro Manzoni 113, Rozzano, MI Italy; 3 Pediatrics, Allergy and Respiratory Disease, Mater Dei Hospital, Belo Horizonte, Brazil; 4 Department of Biomedical Sciences, Humanitas University, Rozzano, MI Italy

**Keywords:** Allergen immunotherapy, Subcutaneous, Children, SLIT, Allergic asthma, Class effect

## Abstract

**Abstract:**

Allergen immunotherapy (AIT) is the only disease-modifying treatment approved for allergic rhinitis and allergic asthma and represents a suitable therapeutic option, especially in childhood, to modify the progression of respiratory allergic diseases. Starting from the previous “generic class effect” evaluation, as testified by the numerous meta analyses, AIT is now considered a product-specific pathogenic-oriented treatment.

**Background:**

AIT was empirically proposed more than one century ago in the subcutaneous form (SCIT), but the IgE-mediated mechanism of allergy was elucidated only after 50 years of clinical use of the treatment. The sublingual administration (SLIT) was developed during the 1980 ties, to achieve an improvement in safety and convenience. While SCIT is approved in the United States for the treatment of asthmatic patients with more than 12 years, so far few trials evaluated the clinical efficacy and safety of SLIT in children with allergic asthma, although the indications and some aspects remain unclear. Certainly, due to compliance problems, the age below 3 years may be reasonably considered a practical contraindication.

**Conclusions:**

Given that some specific AIT products are effective and approved as drugs (AIFA, EMA, FDA), the use in children is still debated. Some aspects still need robust confirm: (a) the safety of AIT in asthma; (b) the optimal regimen of administration; (c) the role of AIT as preventative treatment for asthma development.

## The previous view: the efficacy of AIT as product class

Allergen immunotherapy is the only disease-modifying treatment approved for allergic rhinitis, allergic asthma and hymenoptera venom allergy [1]. Its use is also being investigated for food allergy and atopic dermatitis [2].

Allergen immunotherapy may also represent a valuable therapeutic option, especially in childhood, to modify the progression of respiratory allergic diseases [3]. This remains, indeed, is a unique characteristic of AIT, that is not shared by the standard pharmacological treatments, which only act on symptoms. In fact, AIT intervenes on the pathogenic mechanisms of the allergic disease themselves, by driving the immune system, through the exposure to increasingly higher doses of the etiological allergen(s), to develop a controlled immune response. Thus, so far AIT is the only curative treatment available for allergic diseases approved by the major regulatory authorities in the world including Food and Drug Administration (FDA) and European Medicines Agency (EMA) [4].

Researchers and clinicians are currently facing the era of the so-called “precision” or “personalized” medicine. In such a context, AIT is a good example of personalized therapeutic intervention, in which the most important variables for an appropriate selection of the right therapeutic strategy can be identified [5]. Moreover, in parallel with the refined knowledge of the pathogenic bases of allergic diseases, AIT can guarantee the most appropriate allocation of resources in this time of economical restrictions [6].

Allergen immunotherapy was early proposed in its subcutaneous form (SCIT, subcutaneous immunotherapy) more than one century ago [7]. The sublingual administration (SLIT) was later developed [8], leading to a relevant improvement in safety and convenience, with less frequent severe adverse events. In addition, SLIT allowed the patient’s self-administration with a consequent reduction of the indirect costs.

The efficacy of AIT was grossly evaluated as a “class effect”. This implied that also products with a poorly demonstrated scientific evidence of efficacy were included in meta analyses.

An example of this class effect is a study conducted from June 1999 to June 2000 which enrolled 159 adults and children with allergic asthma with multiple sensitization to grass pollens, tree pollens, weed pollens, animal dander, house-dust mites and moulds. All patients were treated with a standardized aqueous allergen extract in glycerol (ORALVAC, Bencard Allergie GmbH, Munich, Germany) using three concentrations at 100, 1.000 and 10.000 standardized oral units (SOU)/ml. The regimen began with one drop of the lowest concentration and ended with sixteen drops of the highest concentration. Following this incremental phase, a maintenance dose of 16 drops of the 10.000 SOU/ml formulation was administered three times a week for 4–6 months. The results of this study showed a statistically significant reduction in medication for all allergens sensitizations (P = .023) but the asthma “variable” was not highlighted in this study [9].

Several double-blind randomized placebo-controlled clinical trials demonstrated the efficacy of Lais^®^ (Lofarma), a carbamylated allergoid, in patients with HDM-induced allergic rhinitis with or without allergic asthma, proving a reduction in the total and individual symptoms and the drug consumption [10] (Table 1). Another clinical trial showed a reduction in bronchial hyperactivity, nasal inflammation assessed by nasal eosinophil count and asthma and rhinitis symptoms score in patients treated with three different doses of this carbamylated allergoid vs. placebo [11]. Only one clinical trial evaluated Lais administration to 28 children (mean age 13.3 ± 2.1 year) with HDM, Parietaria and Timothy grass-induced allergic rhinitis and/or asthma to verify the occurrence of immediate adverse reactions after an ultra-rush regimen exposure [12]. This study showed an excellent safety and tolerability profile but no other data on Lais in allergic children are at disposition.

**Table 1:** Sublingual immunotherapy preparations available for children so far that have been evaluated as single products in clinical trials

Name	Producer	Composition	Trial population evaluated
Acarizax^®^ (MK-8237)	ALK-Abelló, Hørsholm, Denmark	Standardised allergen extract from house dust mites *Dermatophagoides pteronyssinus* and *Dermatophagoides farinae*	House dust mite-induced Allergic rhinoconjunctivitis with or without coexisting asthma in patients > 18 years old; approved for adolescents in certain countries
Grazax^®^/Grastek^®^	ALK-Abelló, Hørsholm, Denmark	Standardised allergen extract of Timothy grass pollen (*Phleum pratense*)	Grass pollen-induced rhinitis and allergic conjunctivitis in adults and children 5 years or older
Miticure^®^ (MK-8237)	Torii Pharmaceutical Co., Japan)	Extract from the house dust mites *Dermatophagoides (D.) pteronyssinus* and *Dermatophagoides farinae*	Adults and children aged ≥ 12 years
Oralair^®^	Stallergenes, France	Mixture of natural allergens of pollens from five cross-reacting grasses (*Dactylis glomerata* L., *Anthoxanthum odoratum* L., *Lolium perenne* L., *Poa pratensis* L. and *P. pratense* L.)	In Europe and the US for the treatment of grass pollen induced allergic rhinoconjunctivitis in children > 5 years (> 10 years in the US) and adults
Purethal^®^ Mites	Hal Allergy,	Mite extract mixture chemically modified with glutaraldehyde and adsorbed onto aluminum hydroxide	43 asthmatic children (6–14 years) with mites sensitization
Staloral^®^	Stallergenes, France	*D. pteronyssinus* + *D. farinae* extrac	Patients aged 6–18 with allergic rhinitis with or without allergic asthma

Another study conducted from March 2004 to June 2005 evaluated the safety and the tolerability of an ultra-rush method in 100 children (average age of 9.6 years) with allergic rhinitis, asthma, allergic conjunctivitis and atopic eczema. The investigators utilized standardized allergen extract solutions from Anallergo (Florence, Italy) and Staloral 300 from Stallergènes. Final data showed a high level of safety and tolerability for both the preparations [13].

Pollinex^®^ Quattro (Allergy Therapeutics) is an ultra-short course specific immunotherapy (uSCIT) vaccine containing glutaraldehyde-modified allergen extracts formulated with l-tyrosine and with the adjuvant monophosphoryl lipid A (MPL) administered as a pre-seasonal course of four injections. A total of 13 seasonal pollen allergens are extracted and processed to form a series of allergoids with the following dosage scheme: 300, 800, 2000 standardized units (SU) administered in a weekly 1 ml injections, followed by a further top dose injection of 2000 SU to complete the four injections course. Pollinex^®^ Quattro has been evaluated in 3114 patients with multiple sensitization to pollens, 421 of which were children and adolescents 6–18 years with seasonal allergic rhinitis, conjunctivitis and/or asthma. The length of this post-marketing surveillance study was 3 years. AIT was well accepted by children/adolescents and considered very good or good by 93% of the juvenile population. After the first treatment course, antiallergic medication use decreased from 83 to 24% of patients (P < .0001). Response to treatment was assessed as good or very good in 94% of patients and further improvements occurred in patients receiving subsequent courses of therapy [14].

Nowadays, it is recognized that each single product should be evaluated individually, as stated by the World Allergy Organization [15].

It is true that some issues and unmet needs remain critical: (a) at what age should the treatment be started? (b) for how many years does the treatment need to be maintained?; (c) does AIT really prevent the progression of respiratory allergy?; (d) a predictive biomarker of efficacy is required [16].

## AIT in children with allergic asthma

A meta-analysis by Abramson et al. evaluated SCIT in allergic asthmatic patients, both adults and children. Overall, the results showed a significant clinical efficacy based on the standard parameters (symptoms, rescue medications usage, quality of life) [17]. Other available studies on the use SLIT in asthmatic children with mild to moderate HDM-induced asthma showed significant efficacy mainly in the reduction of symptoms and medication usage [18, 19]. The more recent clinical trials in children with birch pollen and grass pollen allergic asthma, further confirmed the efficacy of SLIT in such conditions [20, 21].

According to the more recent evidence, the international asthma documents now recommend a supportive role of AIT, as add on treatment to the standard of care [22]. Uncontrolled asthma remains an absolute contraindication for AIT [23].

The main and relevant aspect of AIT in children is the possible role of the treatment in reducing the risk of asthma onset and of new allergen sensitizations [24]. Children sensitized to one single allergen, as usually happens, when treated with AIT seem to develop new sensitizations at a lesser extent, but this is still matter of debate since the evidence remains poor [25, 26].

On the other hand, there were some randomized open clinical trials (one with SCIT and two with SLIT) showing a preventive effect to decrease the risk of asthma onset in allergic children with rhinitis. This phenomenon was recently confirmed in a randomized and double placebo controlled trial, showing that 2 years after discontinuation of AIT, there was a reduction in symptoms and medication usage [27].

## The current view: evaluation of the “single product”

At present, the efficacy of AIT is no longer considered as a generic “class effect” and each AIT product is evaluated according to its scientific evidence (Table 2). This led to the approval of single AIT preparations by EMA and FDA [28].

**Table 2:** Characteristics of included studies

References	Title	Number of participants and age	Type of immunotherapy (intervention vs. comparator)	Duration of treatment	Author results and conclusions
Valovirta et al. [27]	Results from the 5-year SQ grass sublingual immunotherapy tablet asthma prevention (GAP) trial in children with grass pollen allergy	812 (5–12 years)	SLIT *with Phleum pretense* grass vs. placebo	5 years (3 years treatment and 2 years follow-up)	Treatment with the SQ grass sublingual immunotherapy tablet significantly reduced the risk of experiencing asthma symptoms or using asthma medication (odds ratio = .66, P < .036) and the use of allergic rhinoconjunctivitis pharmacotherapy (27% less, P < .001)
Durham et al. [29]	Sublingual immunotherapy with once-daily grass allergen tablets: A randomized controlled trial in seasonal allergic rhinoconjunctivitis	855 (18–65 years)	SLIT *with Phleum pretense* grass vs. placebo	18 weeks	Moderate reductions of rhinoconjunctivitis symptoms (16%) and medications use (28%). Significantly better rhinoconjunctivitis quality of life scores (P = .006) and an increased number of well days (P = .041)
Ibañez et al. [30]	Safety of specific sublingual immunotherapy with SQ standardized grass allergen tablets in children	60 (5–12 years)	SLIT *with Phleum pretense* grass vs. placebo	1 February 2006 to 3 May 2006	Local reactions were reported at intervention group. The majority of these were local reactions in the mouth or throat and were mostly mild (71%) to moderate (27%) in severity and resolved within days. Two actively treated subjects withdrew from the study: one subject due to four adverse events (moderate eye pruritus, moderate pharyngolaryngeal pain, moderate non-cardiac chest pain and moderate dysphagia) and one subject due to a serious adverse event (asthmatic attack). The subjects recovered completely from the events. SLIT was in general tolerated
Didier et al. [32]	Optimal dose, efficacy, and safety of once-daily sublingual immunotherapy with a 5-grass pollen tablet for seasonal allergic rhinitis	628 (18–45 years)	Standardized 5-grass pollen extract (SLIT) vs. placebo	16 December 2004 to 5 September 2005)	Significantly reduction of rhinoconjunctivitis total score (3.58 ± 3.0, P = .0001; and 3.74 ± 3.1, P = .0006) Compared with placebo (4.93 ± 3.2). The efficacy and safety of sublingual immunotherapy with grass tablets was confirmed
Didier et al. [33]	Sustained 3-year efficacy of pre- and coseasonal 5-grass-pollen sublingual immunotherapy tablets in patients with grass pollen- induced rhinoconjunctivitis	633 (18–50 years)	5-grass pollen tablet (Oralair) vs. placebo	5 years (3-season treatment and 2-year follow-up phases)	The mean symptom score was reduced by 36% and 34.5% at season 3 in the 2 and 4-months pre and coseasonal active treatment groups compared with placebo group (P < .0001 for both). Reductions were observed in total symptom scores and the medication score, with a marked improvement in quality of life for active groups compared with placebo
Lozano et al. [34]	Assessing the Efficacy of Immunotherapy with a Glutaraldehyde-Modified House Dust Mite Extract in Children by Monitoring Changes in Clinical Parameters and Inflammatory Markers in Exhaled Breath	43 (6–14 years)	SIT group (8 μg/ml of Der p1 and 30 μg/ml of Der p2) and treatment group (control group)	Between 2009 and 2011	The SIT group presented with an improvement in asthma classification, a reduction in maintenance drug therapy and improved scores on the quality of-life questionnaire
Ferrés et al. [35]	Efficacy of high-dose sublingual immunotherapy in children allergic to house dust mites in real-life clinical practice	78 (6–18 years)	Retrospective, observational, monocentre study. Medical records of patients who received a standardized *Dermatophagoides pteronyssinus* + *Dermatophagoides farinae* extract (300 IR/ml) were reviewed	Between 2001 and 2008	Patient evaluation of allergy severity revealed a highly significantly improvement between baseline and 6 months (P < .001). This improvement was maintained throughout the 4-year follow-up period. The use of medications was significantly reduced in the first 6 months (4.6 ± 2.5 points at baseline vs. .8 ± 1.6 points at 6 months visit, P < .001) and remained very low until the end of follow-up
Mosbech et al. [40]	Standardized quality (SQ) house dust mite sublingual immunotherapy tablet (ALK) reduces inhaled corticosteroid use while maintaining asthma control: A randomized, double-blind, placebo-controlled trial	604 (14 years or older)	SIT with standardized extracts of *Dermatophagoides pteronyssinus* and *Dermatophagoides farinae* vs. placebo	12 months	Mean difference between 6 SQ-HDM and placebo in the reduction in daily ICS (P = .004). The most common adverse events were local reactions in the mouth
Nolte et al. [41]	Efficacy of house dust mite sublingual immunotherapy tablet in North American adolescents and adults in a randomized placebo-controlled trial	1482 (12 years or older)	SLIT group (tablets with 12 SQ HDM dose) and placebo group	January 2013 to April 2015	Improvement In the total combined rhinitis score by 17% in the SIT group vs. placebo (P = .001). It was well tolerated and improved HDM-induced rhinitis symptoms

*SQ* standardized quality, *SLIT* sublingual immunotherapy, *HDM* house-dust-mite

In 2006 a total of 855 patients with grass pollen induced allergic rhinoconjunctivitis were randomized to placebo or 2.500, 25.000 or 75.000 once daily standardized quality (SQ)—grass allergen sublingual tablets (Grazax^®^/Grastek^®^; ALK-Abelló, Hørsholm, Denmark). This pivotal trial showed a dose-related response with highest reductions in symptoms and medication utilization and improvements in quality of life (QoL) for the 75.000 SQ-T tablet [29]. Further studies confirmed these results in children [30]. Grazax^®^/Grastek^®^ is currently indicated for children > 5 years and adults with allergic rhino conjunctivitis (Table 1).

Oralair^®^ (Stallergenes, France) is a mixture of natural allergens of pollens from five cross-reacting grasses (*Dactylis glomerata* L., *Anthoxanthum odoratum* L., *Lolium perenne* L., *Poa pratensis* L. and *Phleum pratense* L.). Oralair^®^ is approved in Europe and the US for the treatment of grass pollen induced ARC in children > 5 years (> 10 years in the US) and adults with confirming skin prick testing and/or specific IgEs [31].

In 2007 Didier et al. conducted a randomized clinical trial evaluating the dose–response efficacy with a pre-coseasonal Oralair^®^ in 628 patients showing clinical benefits in patients receiving 300 and 500 index of reactivity (IR) 2–4 months before season-start over placebo. Consequently, the 300 IR dose (25 μg of group 5 grass pollen major allergen) providing a significant reduction in symptoms, was selected as the optimal dose for commercialization [32, 33] (Table 1).

Purethal^®^ Mites (HAL Allergy) is a mite extract mixture used at a concentration of 20,000 AUeq/ml (8 μg/ml of Der p1 and 30 μg/ml of Der p2). The administration schedule contemplated a build-up weekly-based phase of increasing doses (1000, 2000, 4000, 6000, 8000 and 10,000 AUeq), whereas the maintenance phase involved the injection of 10,000 AUeq (Der p1, 4 μg of Der p1 and 15 μg of Der p2) at monthly intervals. A total of 43 asthmatic children (6–14 years) with mites sensitization were divided in two groups: 23 individuals were treated with subcutaneous Purethal^®^ for 8 months and 20 were the control group. In the active group, there was an improvement in overall asthma classification and severity (P < .001 compared to baseline), a reduction in drugs use and an improvement in quality of life over time during the follow-up [34].

Staloral^®^ 300 (SLIT with IR (index of reactivity)-standardized *D. pteronyssinus* + *D. farinae* extract (300 IR/mL) manufactured by Stallergenes), has been tested in children aged 6–18 with allergic rhinitis with or without allergic asthma [27]. All patients treated with Staloral^®^ reported improved rhinitis and/or asthma symptoms scores, together with a reduction in the as-needed drug for breakthrough symptoms, with no significant differences in FEV1 and Peak Flow [35] (Table 1).

Another house dust mite sublingual preparation (MK-8237) has been approved for patients with HDM-induced ARC with or without coexisting asthma [36] (Table 1).

The product, a standardized allergen extract from *D. pteronyssinus* and *D. farinae* 12 SQ-HDM* per oral lyophilisate, has been approved in 2015 by EMA with the brand name of Acarizax^®^ for Europe, Canada and Australia (ALK-Abelló, Hørsholm, Denmark), and it is the first SLIT preparation approved in Europe for the treatment of allergic asthma. In fact, in February 2017 the Global Initiative for Asthma (GINA) for the first time added AIT as a treatment option in its guidelines [22]. Originally approved only for patients > 18 years old, Acarizax^®^ was recently approved also for adolescent patients with house dust mite-induced allergic rhinitis in Austria, Czech Republic, Denmark, Finland, Italy, the Netherlands, Norway, Poland, Sweden, Slovakia, France and Germany [37].

MK-8237 has been approved in September 2015 by the Japanese Ministry of Health, Labour and Welfare under the name of Miticure^®^ (Torii Pharmaceutical Co., Japan), at a dose of 3300 Japanese Allergen Units (JAU) once a day during the 1st week of treatment and one tablet of Miticure^®^ 10,000 JAU once a day from the 2nd week for adults and children aged ≥ 12 years [38] (Table 1). Nowadays, since there are no indications of MK-8237 utilization in children so far, the Company is running a Phase III clinical trial in patients aged 5–11 years [39].

The efficacy of this preparation in asthmatic patients was demonstrated in three clinical trials, two of which included also adolescents.

Mosbech and colleagues, enrolled 604 subjects including adolescents aged > 14 years with HDM-induced allergic rhinitis and mild-to-moderate asthma. These subjects were randomized in 1:1:1:1 to double-blind daily treatment with active doses (1, 3, or 6 SQ—HDM) or placebo. The main outcome was a steroid-sparing effect which resulted dose-dependent, higher in subjects receiving 6 SQ HDM-SLIT tablets and that mild-to-moderate HDM-sensitized asthmatic patients could significantly benefit from specific immunotherapy [40]. Furthermore, MK-8237 was evaluated in a recent north American clinical trial (NCT01700192) in adolescents aged 12 years old with HDM-induced allergic rhinoconjunctivitis with or without asthma. This trial confirmed the results obtained by the previous European trials [41].

So far, only one trial to evaluate the clinical efficacy and safety of SLIT in children is still in progress [39]. Thus the need for more robust data in such a population is emergent.

## Indications and patients’ selection

Definite indications for the use of AIT in children with asthma are still not fully clarified [16]. Most guidelines [24] agree that AIT is not contraindicated in children with mild to moderate allergic asthma, but also state that asthma must be fully controlled by the standard of care pharmacological treatment, when AIT is prescribed [16, 17, 42, 43]. The recent ARIA guidelines [44] suggest both SCIT and SLIT as a conditional recommendation in allergic asthma, due to the moderate/low quality of evidence. The lack of robust evidence led to a certain grade of opposition to the use of AIT in asthmatics [45]. Indeed, the potential benefits of AIT must be weighed against the risk of adverse effects, the inconvenience and cost of a prolonged course of therapy, as stated by the 2017 Global Initiative for Asthma Report [46] considering also other factors such as poor adherence, clinically non relevant allergens, poly-sensitizations, unavoidable adverse reactions of routine medication, etc [47].

## Contraindications

So far, according to literature, absolute contraindications to AIT involve serious immunologic diseases, major cardiovascular diseases, cancer, chronic infections, lack of compliance and severe psychological disorder [23]. Eosinophilic esophagitis remains an absolute contraindication to SLIT [48].

Relative contraindications include any condition that would reduce the patient’s ability to survive a potential systemic allergic reaction [42]. Temporary contraindications are limited to acute infections and, for SLIT, to oral acute inflammation, injury or surgical intervention or acute gastroenteritis [4, 48–50].

Well-controlled asthma, independently from its severity, is not an absolute contraindication to AIT [23], whereas uncontrolled asthma is an absolute contraindication due to the risk of serious or even fatal adverse reactions [23, 47, 51]. In this context, the pre-treatment with omalizumab [52] has been suggested as a suitable option to increase the safety. Partially controlled asthma is considered a relative contraindication in the EAACI paper and the German guidelines open to the possibility of use of AIT in these patients [48]. An open area of debate regards well controlled severe asthma, which would not meet the EAACI definition of contraindication, but for which more evidence should become available before recommendations can be issued.

## Age of AIT initiation

The age of 3 years is considered a reasonable contraindication, due to the poor adherence and side effects [53] and age below 5 years is listed as a relative contraindication to AIT in most documents [23, 49, 54], although there are positive reports in such age range [55–58]. The reasons for this choice can be found in the limited evidence [59] and for practical reasons, but this issue may deserves more in-depth studies in the perspective of preventive strategies. In fact, a recent position paper by the SIAIP (Società Italiana di Allergologia e Immunologia Pediatrica) [43] recommends the consideration of AIT as a therapeutic option also in preschool age.

## Administration regimen, duration of treatment and adherence

While there is a universal consensus about administration regimen for SCIT, with the maintenance phase given as one injection every 4 weeks, the best SLIT maintenance regimen has not been defined yet [16]. The recommended regimens would favour the pre-coseasonal administration vs. the continuous one, at least for seasonal allergens [60, 61].

It is usually recommended that at least 3 years of treatment are necessary to achieve and maintain clinical benefit [4, 48–50]. A prolonged remission of symptoms is described in many patients [62–66], while others may experience a relapse after discontinuation. As for adults, it is still not clear in pediatric populations whether treatment continuation beyond 3 years leads to persistence of clinical benefit after discontinuation [29, 62, 64, 67] and if other factors are involved. The decision about the duration of treatment remains on the clinician judgment, who should take into account the patient clinical response, disease severity, adverse events and patient’s preference [42]. In fact, adherence to therapy is an important issue, due to the long duration of treatment [68]. It was shown that 64.6% of children was unable to complete 3 years of therapy [69]. The reported major factors involved in non-compliance were the cost of treatment, the inconvenience of injections for SCIT and the daily necessity of assumption for SLIT and local reactions [70, 71].

## Safety

Adverse reactions to AIT can be divided in local (limited to the site of administration) [72] and systemic (wheezing, urticaria, anaphylaxis, fatal reactions) [17]. Local reactions consist mainly in local itching and/or edema and, only for SLIT, gastrointestinal complains [46].

Systemic reactions are more frequent in SCIT, for which the reported incidence varies between .06 and 1.01% [73], whereas with SLIT systemic reactions are reported to occur quite rarely [74].

In a recent prospective European survey [75] involving 762 children and 801 adolescents undergoing AIT, a total of 29 reactions have been reported, of which 23 during SCIT and 6 during SLIT, comprising 3 cases of anaphylaxis, all related to SCIT. Interestingly, the reported cases of anaphylaxis all had a delayed onset (> 2 h after administration), which highlights an open issue about the correct duration of patient observation after AIT administration, that should last at least 30 min according to current recommendations [49, 50]. Sublingual administration appears to be correlated with a much lower risk of systemic reactions. A 2009 observational study in 193 children receiving SLIT [76] reported nearly 500 mild local reactions and only one systemic reaction consisting in a severe asthma attack and a recent review of 80 double-blind placebo-controlled trials concluded that in most studies the incidence of systemic reactions was similar in the treatment group and the placebo group [46].

The occurrence of adverse reactions depends on several factors, including human errors, extracts used, administration route, schedule and the dose administered [52]. Patient-related factors comprise, as already stated, the presence of asthma, especially if uncontrolled [23], polysensitization and grass pollen sensitization [75]. The administered dose seems to be related to systemic reactions only in SCIT, while this does not appear to be dose-dependent with SLIT [47].

## Conclusions and expert opinion

Allergen immunotherapy remains a cornerstone option for the treatment of respiratory allergy and for hymenoptera venom allergy with a promising extension to food allergy. AIT especially in the sublingual administration, remains a suitable option in children since it can be easily managed at home, although some aspects still need to be experimentally defined [77]. The possible use of AIT as primary prevention still remains a matter of debate, whereas the clinical efficacy in children is well ascertained, at least for some specific products [78]. Asthma, when well controlled does not represent an absolute contraindication to AIT. There is a consistent evidence that AIT can reduce the risk of asthma onset in sensitized children.

## Abbreviations

AIT: allergen immunotherapy
FDA: Food and Drug Administration
EMA: European Medicines Agency
SCIT: subcutaneous immunotherapy
SLIT: sublingual immunotherapy
AR: allergic rhinitis
AA: allergic asthma
HDM: house-dust-mite
GINA: Global Initiative for Asthma
ICS: inhaled corticosteroids
ARC: allergic rhinoconjunctivitis
SQ: standardized quality
SABA: short acting beta-agonist
LABA: long acting beta-agonist
ARIA: Allergic Rhinitis and its Impact on Asthma
EAACI: European Academy of Allergy and Clinical Immunology
